# Indirect effects shape macroalgal epifaunal communities

**DOI:** 10.1002/ece3.8195

**Published:** 2021-10-09

**Authors:** Aldwin Ndhlovu, Justin A. Lathlean, Christopher D. McQuaid, Laurent Seuront

**Affiliations:** ^1^ Department of Zoology and Entomology Rhodes University Grahamstown South Africa; ^2^ Department of Zoology Nelson Mandela University Gqeberha South Africa; ^3^ LOG Laboratoire d'Océanologie et de Géosciences CNRS, UMR 8187 Univ. Lille Univ. Littoral Côte d'Opale Wimereux France; ^4^ Department of Marine Resources and Energy Tokyo University of Marine Science and Technology Tokyo Japan

**Keywords:** ecosystem engineer, epifauna, fractals, *Gelidium pristoides*, habitat, rocky shore, upwelling

## Abstract

We tested the response of algal epifauna to the direct effects of predation and the indirect consequences of habitat change due to grazing and nutrient supply through upwelling using an abundant intertidal rhodophyte, *Gelidium pristoides*. We ran a mid‐shore field experiment at four sites (two upwelling sites interspersed with two non‐upwelling sites) along 450 km of the south coast of South Africa. The experiment was started in June 2014 and ran until June 2015. Four treatments (predator exclusion, grazer exclusion, control, and procedural control) set out in a block design (*n* = 5) were monitored monthly for algal cover for the first 6 months and every 2 months for the last 6 months. Epifaunal abundance, species composition, algal cover, and algal architectural complexity (measured using fractal geometry) were assessed after 12 months. Predation had no significant effect on epifaunal abundances, while upwelling interacted with treatment. Grazing reduced the architectural complexity of algae, with increased fractal dimensions in the absence of grazers, and also reduced algal cover at all sites, though the latter effect was only significant for upwelling sites. Epifaunal community composition was not significantly affected by the presence of herbivores or predators but differed among sites independently of upwelling; sites were more similar to nearby sites than those farther away. In contrast, total epifaunal abundance was significantly affected by grazing, when normalized to algal cover. Grazing reduced the cover of algae; thus, epifaunal abundances were not affected by the direct top‐down effects of predation but did respond to the indirect effects of grazing on habitat availability and quality. Our results indicate that epifaunal communities can be strongly influenced by the indirect consequences of biotic interactions.

## Graphical abstract

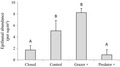

## 1 INTRODUCTION

Despite an early emphasis on top‐down forcing in marine ecosystems (e.gConnell, 1970; Paine, 1974) and great recognition of bottom‐up forcing in terrestrial systems (e.gChen & Wise, 1999; White, 1978), there now appears to be a broad consensus that in both terrestrial (Daughtery et al., 2007; Hunter & Price, 1992) and marine (Menge, 2000; Worm et al., 2002) systems, populations are regulated by a combination of bottom‐up (resource availability) and top‐down (predation) effects, with the balance between the two differing across space and time and across gradients of environmental stress. Importantly, in marine systems a range of bottom‐up effects are driven by water movement, including the supply of nutrients, suspended food particles, and larvae (Menge, 2000). For example, large‐scale phenomena such as upwelling affect nutrient supply to primary producers (Andrews & Hutchings, 1980; Pitcher et al., 1991), but very small‐scale effects such as guano input (Methratta, 2004) or even epifaunal excretion (Probyn & Chapman, 1983; Taylor & Rees, 1998) can also be important at local scales. The effects of bottom‐up forcing mechanisms such as upwelling can, however, be strongly context‐dependent (Blanchette et al., 2009), with rates of bottom‐up supplies influencing top‐down species interactions (Menge et al., 2003).

The existence of trophic cascades is an indication that top‐down effects can be very strong yet indirect. Examples include the collapse of populations of a top predator, cod, that resulted in high abundances of the planktivorous Sprat, which then hindered the recovery of cod by preying on their larvae (Casini et al., 2009). Another well‐known example is the decline of kelp forests in North America due to the increase in sea urchin grazing following reductions in urchin predators (Tegner & Dayton, 1991). In the Aleutian Islands, this decline has further been linked to the top‐down control of sea otter population by killer whales, with subsequent cascading positive and negative effects on sea urchins and kelp (Estes et al., 1998). Top‐down effects are typically trophic, but we wished to test the possibility that they could lead to indirect, non‐trophic effects, specifically through their influence on habitat availability and quality, and how this might interact with the direct top‐down effects of predation. To test this, we examined how the abundance and community structure of macroalgal epifauna are affected by the direct top‐down effects of predation and the indirect consequences of top‐down and bottom‐up effects on the availability and quality of macroalgal habitat. We studied the rhodophyte *Gelidium pristoides*, a commercially harvested macroalga (Anderson et al., 1989) in South Africa that dominates algal cover on the mid‐shore. It usually comprises separate tufts and grows to a maximum of about 15 cm in length (Gibbons, 1988). *Gelidium pristoides* generally experiences high levels of grazing by macrograzers, particularly from a range of limpets such as *Siphonaria* spp. and *Cymbula oculus*, as well as various snails (Branch, 1981).

Macrophytes such as *Gelidium* spp. are important as primary producers and as ecosystem engineers providing habitat for a wide range of organisms (Cattaneo & Kalff, 1980). These include meiofauna (Gibbons & Griffiths, 1986) and high densities of epifaunal invertebrates, mostly polychaetes, small crustaceans, and gastropods. These species use macroalgae for protection from predators (e.g., Kon et al., 2009; Machado et al., 2019), mainly fish in our system (Newcombe & Taylor, 2010), and can feed on the macroalgae directly (Duffy, 1990) or feed on the periphyton they support (Brawley & Fei, 1987; Klumpp et al., 1992). The species composition of invertebrate communities can differ among algal species, and this appears to be related to the physical architecture of the algae including the structure, design, and organization of the algae rather than their species identity (Duffy et al., 2001; Taylor & Cole, 1994).

Growth rates of macroalgae respond directly to increased nutrient availability provided through upwelling or eutrophication (e.gNielsen & Navarrete, 2004; Worm & Lotze, 2006), and in areas with high nutrient input, they can strongly shape intertidal communities. For example, rocky shores in upwelling regions support significantly greater algal cover, abundances of sessile organisms, and biomass of herbivorous limpets per unit area than shores in regions lacking coastal upwelling (Bosman et al., 1987). Intertidal macroalgae have frequently been shown to be controlled by grazing (Duffy & Hay, 2001; Hawkins & Hartnoll, 1983), but the effects of grazing in intertidal systems can result in patchy distributions of macroalgae that can show marked spatial determinism (Diaz & McQuaid, 2011). In the case of epifaunal communities, macroalgae provide a point of attachment and protection from predation during high tide and a refuge from heat and desiccation stress during low tide (Wright et al., 2014). By reducing habitat availability, the top‐down effects of grazing on macroalgae could have significant indirect consequences for epifaunal abundance and community structure. Similarly, macroalgal growth rates respond directly to increased nutrient availability so that the direct bottom‐up effects of nutrient input on macroalgal growth and abundance are likely to have indirect consequences for the associated epifauna.

In this context, we used manipulative experiments to test the following hypotheses:

1. Predation has direct top‐down effects on epifaunal abundances.
2. The effects of predation are stronger where upwelling is less frequent and less intense.
3. Top‐down (grazing) and bottom‐up (upwelling) effects on macroalgae will have indirect consequences for epifaunal communities.

## 2 METHODS

**FIGURE 1:**
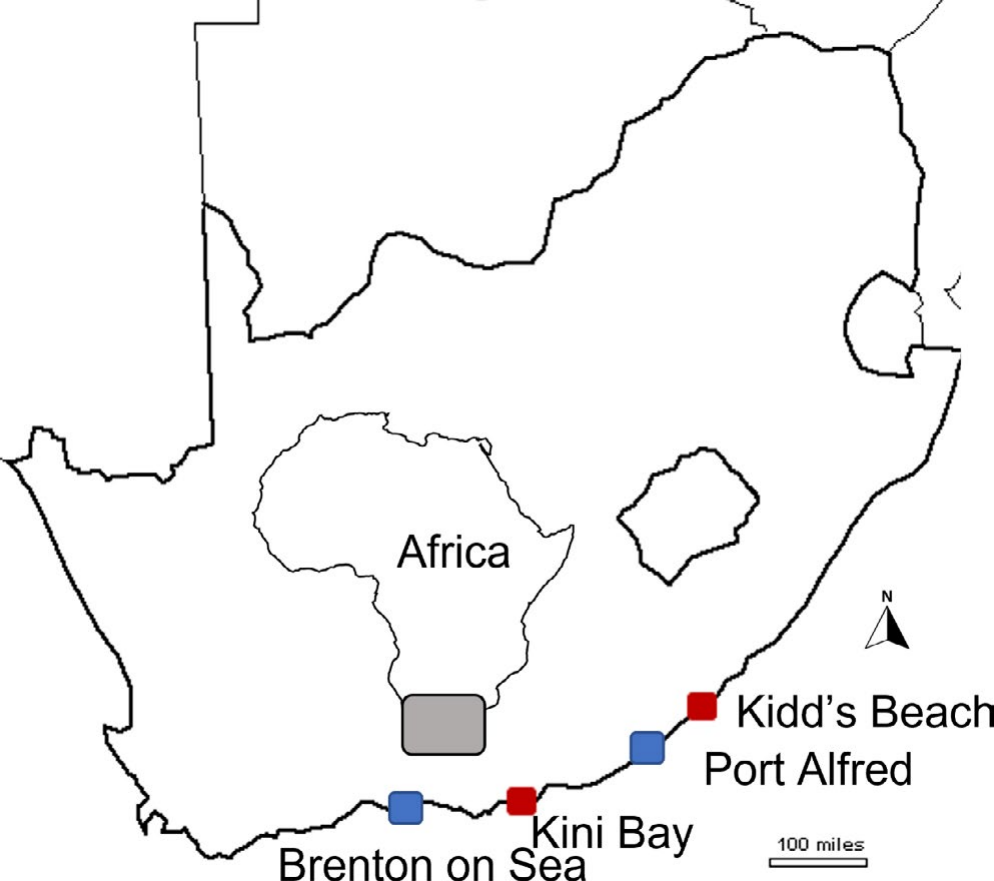
Map showing all the four study sites in the southeast coast of South Africa. Red representing non‐upwelling and blue upwelling sites

This study was undertaken at four moderately exposed rocky shores, separated from each other by 10 s of km along c.500 km of the south coast of South Africa (Figure 1). Two rocky shores were designated a priori based on the available literature as upwelling sites (Port Alfred: 34°36′85.8″S, 26°53′55.8″E; and Brenton‐on‐Sea: 34°04′31.7″S, 23°01′29.5″E) and were interspersed with two non‐upwelling sites (Kidd's Beach: 32°55′14.2″S, 27°29′18.0″E; and Kini Bay: 34°01′17.2″S, 25°22′58.3″E). Apart from upwelling, all sites exhibit similar environmental conditions, including slope, tidal range, degree of wave exposure, and orientation toward the incoming waves, and support similar benthic communities, with the mid‐shore regions being characterized by monospecific beds of the rhodophyte *G. pristoides*.

### 2.1 Characterizing upwelling

After subjectively categorizing the study sites as upwelling or non‐upwelling, we confirmed this using two complementary approaches.

#### 2.1.1 In situ temperature data

To gauge the frequency and intensity of upwelling, three temperature iButtons (DS1921L model; Dallas Semiconductor), embedded in a waterproof resin (3 M Scotchcast 2130 Flame Retardant Compound), were deployed in the mid‐intertidal zone at each site to record both air and sea temperatures with a resolution of 0.06°C and an accuracy of 0.5°C every 30 min from September 2014 to June 2015. Temperatures for each site were analyzed using the average of the three data iButtons. Tidal data were used to identify periods of logger submergence, and the number and duration of upwelling events were estimated by identifying periods when sea temperatures dropped by 5°C or more within 24 h. Upwelling duration was estimated as the number of days it took for temperature to return to the temperature before the onset of the upwelling event.

#### 2.1.2 Wind data

Wind speed and direction were used to characterize coastal upwelling. We used wind data to calculate an upwelling index, which was used to quantify the duration and intensity of upwelling events at our four study sites. Wind data for the duration of the experiment were collected from four meteorological stations, each within 10 km of one of the four sites (South African Weather Service, 2015). These were as follows: East London, Port Alfred, Port Elizabeth, and Knysna. Hourly wind speed and direction for each day were used to calculate an upwelling index (UPW) following Bakun (1975) as:

$$\text{UPW}=\rho_{a}\times C_{d}\times v\times \vec{v}\times f^{‐1}\times \rho_{w}^{‐1}$$

where *ρ_a_* is the air density, *C_d_* is the drag coefficient (i.e., ca. 0.0014), *v* is the mean height‐corrected wind speed, $\vec{v}$ is the alongshore vectorial component (estimated as zonal winds from the study sites), *f* is the Coriolis frequency (*f* = 9.9 × 10^−5^ rad s^−1^) at middle latitudes, and *ρ_w_* is the water density (i.e., *ρ_w_* = 1025 kg m^−3^). Positive values represent periods of upwelling, and negative values represent periods of downwelling. The duration of upwelling events was categorized as long (≥6 days), medium (3–6 days), or short (≤3 days).

### 2.2 Experimental design

Twenty stainless steel cages (20 × 20 × 15 cm; mesh size 20 mm) were screwed on the rocks in the mid‐shore region of each site where abundances of *G. pristoides* were high to moderate and grazer abundances were similar (A. Ndhlovu, pers. obs). Treatments were distributed in a random block design with a total of five blocks separated by 5–10 m. Treatments (cages and control plots) were separated by 1 to 2 m within each block. Each block included four treatments: (i) total exclusion cages (TE), roofed cages that excluded both benthic grazers and pelagic predators; (ii) partial exclusion cages or grazer‐only cages (G+), roofed cages with sides that did not reach the substratum, allowing access to benthic grazers but not pelagic predators; (iii) open roof or predator‐only cages (P+), cages with closed sides and an open roof allowing access to pelagic predators, but not benthic grazers; and (iv) control areas (Co), which had screws marking the four corners of the plot but were otherwise unaltered. Preliminary tests were conducted to infer potential cage artifacts, such as the effects of altered hydrodynamics and/or light availability on algal growth. Observations carried out before the beginning of the experiment confirmed the effectiveness of the treatments. Grazers such as the limpet *Siphonaria concinna* were observed within the grazer‐only cages and control plots but were very rarely observed in the total exclusion or predator‐only cages. Cages were regularly checked throughout the experiment and on the few occasions when grazers were found in inappropriate treatments they were removed. The experiment ran from June 2014 to June 2015 with sampling each month for the first 6 months and every two months thereafter. During each sampling event, photographs were taken of algal cover in each plot and a wire brush was used to remove any algae growing on the cages. Because *G. pristoides* was the only alga present in the plots, there was no problem of algal overgrowth and percentage algal cover within plots could be calculated from the photographs using Coral Point Count (CPCe) and the point intercept method (Kohler & Gill, 2006; Lathlean et al., 2015).

After 12 months, the cages were removed and all *G. pristoides* and the associated epifauna were collected and stored in 10% formalin before subsequent sorting took place. In the laboratory, algal samples were washed of all organisms before the blotted weight and the dry weight of *G. pristoides* were measured. Blotted weight was taken immediately after washing with the *G. pristoides* rolled in tissue paper. Dry weight was measured after drying at 60°C for 48 h. All organisms washed from the algae were stored in 70% ethanol and identified to species level.

The architectural complexity of *G. pristoides* was assessed using fractal geometry (Mandelbrot, 1983), a mathematical tool that can be used to describe the structure of complex objects in situations where Euclidean descriptors are inappropriate (Seuront, 2010; Sugihara & May, 1990). For each treatment, five individual *G. pristoides* were photographed from four different angles, each picture being taken at right angles to the preceding one to capture the three‐dimensional structure of the algae. The fractal dimension, *D_i_*, was subsequently estimated for each photograph *i* by superimposing a regular grid of squares of size *l* on the image of the algae and counting the number of “occupied” squares. This procedure was repeated using different values for *l*. The surface occupied by the algae was then estimated with a series of counting squares spanning a range of surfaces down to a small fraction of the entire surface. In the presence of a fractal structure, the number of occupied squares increases with decreasing square size, leading to a power‐law relationship of the form $N\left(l\right)=kl^{{‐D}_{i}}$, where *l* is the box size, *N*(*l*) is the number of squares occupied by the image of the algae, *D_i_* is the so‐called fractal dimension, often referred to as the box‐dimension, and *k* is a constant (Seuront, 2010). *D_i_* is estimated from the slope of the linear trend of the log–log plot of *N*(*l*) vs. *l*, that is, log *N*(*l*) = log *k* − *D_i_* log *l*. Because slight reorientation of the overlying grid can produce different values of *N*(*l*), *D_i_* was estimated for rotation of the initial 2D grid of 5° increments from 0 to 45° (Seuront et al., 2004). The potential presence of anisotropy in the structural complexity of *G. pristoides* was assessed through a comparison of the fractal dimensions *D_i_* using an analysis of covariance (Zar, 1999). If the null hypothesis of nonsignificant differences in *D_i_* between replicates was rejected, the data from the four replicates were pooled and a common fractal dimension *D* was used in further analysis after successfully inferring the lack of statistical significance between intercepts (Zar, 1999).

### 2.3 Data analysis

Data used to distinguish between upwelling and non‐upwelling sites fulfilled the prerequisites for parametric analysis (Shapiro–Wilk test and Levene's test) and were analyzed using one‐way ANOVA. Data for algal cover similarly fulfilled the prerequisites for parametric analysis and were analyzed using a three‐way nested ANOVA to assess the influence of treatment (fixed, four levels), upwelling (fixed, two levels), and site (nested in upwelling, random, four levels) on percentage algal cover. This was done at the start of the experiment (no significant effects of any factor or interaction) and again toward the end of the experiment. The site at Brenton‐on‐Sea was briefly inundated with sand after 9 months, between February and March 2015 so this analysis was run on the data for February. Analyses of the epifauna were based on data collected at the end of the experiment when all plots were destructively sampled. We used the same nested design to test the effects of treatment, upwelling, and site on: epifaunal abundance (i.e., total number of epifaunal individuals) in each plot and density of epifauna per unit algal cover (number of epifauna per cm² of algal cover) in each plot. Analyses were performed using Statistica 12 (StatSoft), with alpha = 0.05.

Epifaunal community structure was analyzed by three‐way type III permutational analysis of variance (PERMANOVA) with 9999 permutations based on the Bray–Curtis similarity matrix to test the effects of treatment, site, and upwelling. To visualize the results, non‐metric multidimensional scaling (nMDS) ordinations based on the Bray–Curtis similarity matrix measures with untransformed data were plotted. SIMPER analysis was used to assess the percentage contributions of each species to differences among sites. Analyses were done using PRIMER 6.

Because fractal dimensions *D* were non‐normally distributed (*p* > .05), multiple comparisons between treatments were conducted using the Kruskal–Wallis test, and a subsequent multiple comparison procedure based on the Tukey test was used to identify distinct groups of measurements (Zar, 1999).

## 3 RESULTS

### 3.1 Upwelling

**FIGURE 2:**
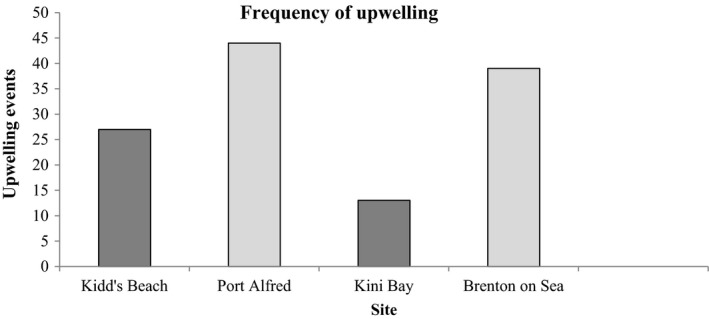
Total number of upwelling events at all four sites (Kidd's Beach, Port Alfred, Kini Bay, and Brenton‐on‐Sea)

The two upwelling sites, Port Alfred and Brenton‐on‐Sea, experienced more persistent and more frequent upwelling events (44 and 39, respectively) than Kidd's Beach and Kini Bay (27 and 13 events, respectively; Figure 2) although intensity, measured as mean upwelling index, was lowest at Brenton‐on‐Sea (Table 1).

**TABLE 1:** Upwelling events recorded for each site

Period	Kidd's Beach	Port Alfred	Kini bay	Brenton‐on‐Sea
Upwelling index				
Jun–August	8	13	13	15
Sep–Dec	18	31	21	31
Jan–April	21	26	21	27
May–Jun	7	9	7	7
Total	54	79	62	80

### 3.2 Algal cover and complexity

**FIGURE 3:**
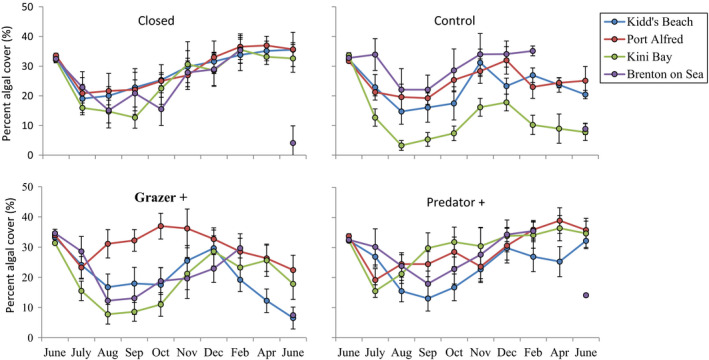
Mean percentage algal cover in the Closed, Control, Predator +, and Grazer + plots. Values are means plus/minus standard deviation. Brenton‐on‐Sea was inundated with sand after February so that data are missing for April

**FIGURE 4:**
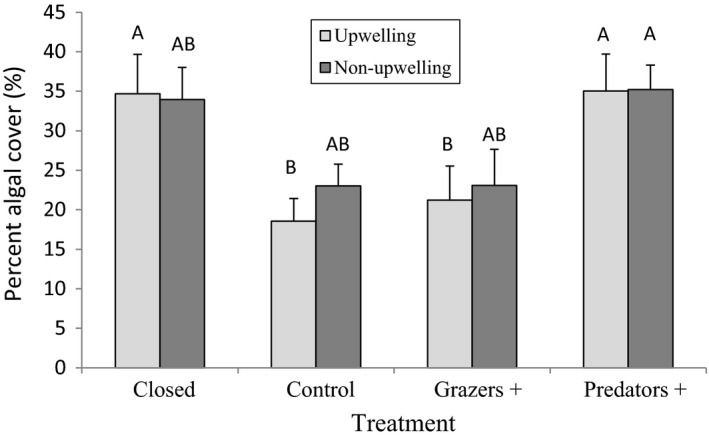
Mean algal cover of different treatments for upwelling sites (Port Alfred and Brenton‐on‐Sea) and non‐upwelling sites (Kidd's Beach and Kini Bay). Shared letters indicate groups that are not significantly different (ANOVA, *p* = .05)

Overall algal cover varied with season across all treatments, being greatest in summer and lowest in winter, but changes in cover throughout the course of the experiment differed among the four experimental treatments at all study sites. These differences were most pronounced in July–September (Figure 3). Upwelling regime had no significant effect on cover of *G. pristoides* after 10 months (i.e., immediately before Brenton‐on‐Sea was inundated by sand in March) (ANOVA: *F*1,67 = 0.65, *p* > .05; Table 2). Cover did, however, differ among treatments, but the nature of such differences differed among sites (Treatment × Site interaction, ANOVA: *F*3,67 = 4.56, *p* < .05; Table 2). For example, at all sites, treatments allowing access to grazers (Control and Grazer +) showed lower algal cover than those excluding grazers (Closed and Predator +), suggesting strong grazing pressure, but the difference was only significant at the two upwelling sites (Port Alfred and Brenton‐on‐Sea) (Figure 4, Tukey HSD). This reflects the fact that grazer numbers are significantly higher at upwelling sites (unpubl. data).

**TABLE 2:** Summary statistics of three‐way nested ANOVA to assess the influence of treatment (fixed, 4 levels), upwelling (fixed, 2 levels), and site (nested in upwelling, random, four levels) on (i) percentage algal cover, (ii) epifaunal abundance, and (iii) density of epifauna per unit algal cover. Post hoc analyses represent Tukey's HSD tests

Response variable	Df	MS	*F*	*p*
Percentage algal cover				
Upwelling	1	50.75	0.65	.57
Site [Upwelling]	1	78.61	2.27	.14
Treatment	3	618.7	3.91	.15
Upwelling × Treatment	3	197.2	5.68	.**001**
Site [Upwelling] × Treatment	3	158.23	4.56	.**01**
Error	67	34.7		
Post hoc: see main text				
Epifaunal abundance				
Upwelling	1	67448.4	3.99	.3
Site [Upwelling]	1	16889.2	4.79	.**03**
Treatment	3	3487.3	0.93	.52
Site [Upwelling] × Treatment	3	9418.7	2.67	.06
Upwelling × Treatment	3	3729.7	1.06	.38
Error	51	3525.6		
Post hoc: *p* < .05 in all cases				
Density of epifauna per unit algal cover				
Upwelling	1	6.8128	11.54	.18
Site [Upwelling]	1	0.5903	0.15	.7
Treatment	3	38.5508	17.71	.**02**
Site [Upwelling] × Treatment	3	1.8634	0.47	.7
Upwelling × Treatment	3	2.1767	0.55	.65
Error	51	3.9302		
Post hoc: Control = Grazer + ≠ Predator + = Closed				

**FIGURE 5:**
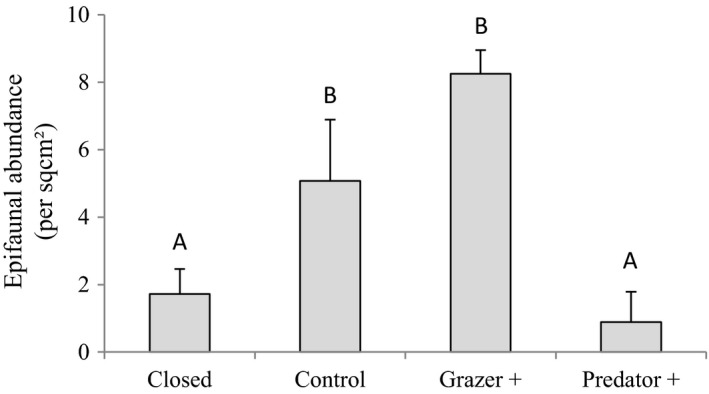
Mean fractal dimension among treatments (ANOVA, *p* = .05)

The structural complexity of *G. pristoides* was consistently described in terms of fractals. Specifically, no significant differences were found between the fractal dimensions *D_i_* estimated from each replicate photograph of each individual *G. pristoides*. This indicates an absence of anisotropy in the structural complexity of *G. pristoides*. The fractal dimensions *D* significantly differed among treatments (*p* < .01), indicating increased complexity in the order *D*_Control_ < *D*_Grazer only_ < *D*_Predators only_ = *D*_Caged_ (Figure 5).

### 3.3 Epifaunal community structure

Each site supported between 13 and 19 species of epifauna, with an overall total of 44 species identified across the four sites. The most abundant taxa were crustaceans (amphipods and isopods), with some polychaetes and gastropods (Table A1).

PERMANOVA of raw data and of data normalized for algal cover showed that neither upwelling nor treatment had a significant influence on the community structure of epifaunal communities, but site did (PERMANOVA, *p* < .0001 in both cases) (Tables 3 and 4).

**TABLE 3:** Percentage dissimilarities in species composition among sites

	Kidd's Beach	Port Alfred	Kini Bay	Brenton‐on‐Sea
Kidd's Beach				
Port Alfred	75.47			
Kini Bay	74.03	66.32		
Brenton‐on‐Sea	85.73	83.41	84.82	

**TABLE 4:** Summary of results between Grazer + vs Closed and Predator + vs Control plots when data were normalized for algal cover

	Normalized to cover
Grazer + vs Closed	*p* < .05
Predator + vs Control	*p* < .05

The influence of site was partially due to geography, with the SIMPER analysis indicating that sites that were farther apart were generally more dissimilar than sites that were close to one another.

### 3.4 Epifaunal abundance

Generally, comparisons of treatments produced different results for total abundances and data normalized for algal cover. The key tests for the effects of predation on total epifaunal abundance were the comparison of Control vs Grazer + plots and of Closed vs Predator + plots, with both comparisons being nonsignificant.

#### 3.4.1 Total abundance

The only significant effect on total epifaunal abundances within plots was site (*p* = .033; Table 2). All sites were different from each other (Tukey's HSD, *p* < .05 in all cases). Total epifaunal abundance declined in the order Port Alfred > Kidd's Beach > Kini Bay >Brenton‐on‐Sea.

#### 3.4.2 Normalized to algal cover

**FIGURE 6:**
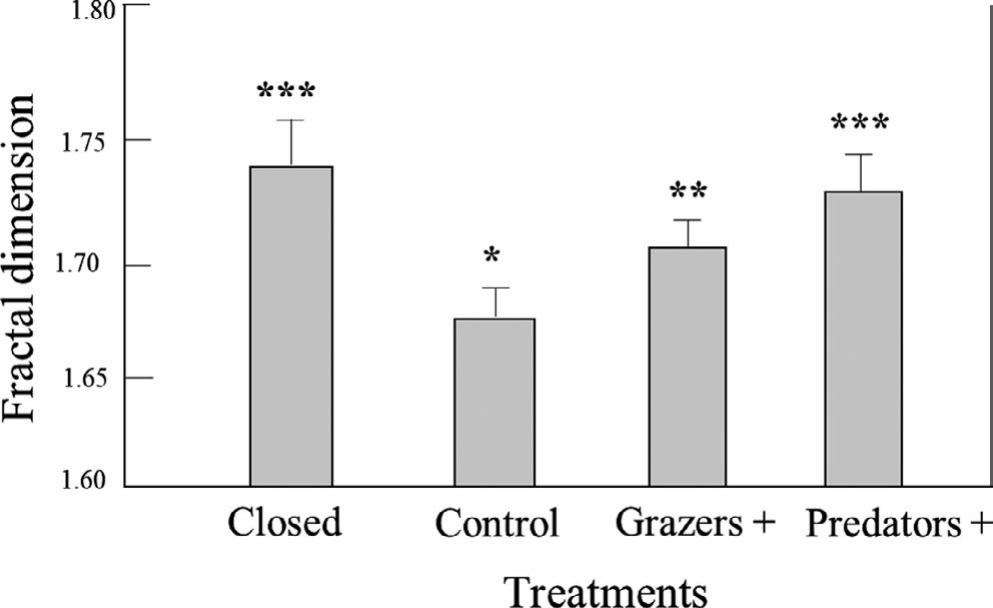
Mean (± SE) epifaunal abundance normalized to algal cover and among treatments pooled across all four locations. Shared letters indicate groups that are not significantly different (ANOVA, *p* = .05)

When epifaunal abundances were normalized to unit area of algal cover, the only significant effect was treatment, with significantly lower numbers within treatments where grazers and predators were excluded (Tukey's HSD, *p* < .05; Table 2). Interestingly, plots that were open to grazers (i.e., Controls and Grazer +) supported higher normalized abundances even though the percentage cover of *G. pristoides* was found to be negatively affected by the presence of grazers (Figure 6). This means that grazing reduced algal cover, but not total epifaunal abundances, resulting in higher densities per unit of algal cover.

### 3.5 Summary of results

1. Predation—no effects on epifaunal abundances in the presence or absence of grazers. Generally, comparisons of treatments produced different results for total abundances and data normalized for algal cover. The key tests for the effects of predation on epifaunal abundance were the comparison of the Control vs Grazer + plots and Closed vs Predator + plots, with both comparisons being nonsignificant.
2. Grazing—It reduced the cover of *G. pristoides* (though the effect was significant only at upwelling sites) and its architectural complexity but had no effect on total epifaunal abundances. Together, these effects of grazing resulted in a significant increase in epifaunal densities.

## 4 DISCUSSION

*Gelidium pristoides* is an economically and ecologically important seaweed; it influences the structure and functioning of epifaunal communities and can be viewed as a keystone species, foundation species, or ecosystem engineer. *Gelidium pristoides* is responsible for influencing the composition and abundance of other species in the community (Jones et al., 1997; Shelton, 2010) by modifying environmental conditions, relationships between species, and the availability of resources. Ecosystem engineers can be used to assess the likelihood of successful ecosystem restoration (Byers et al., 2006) as they can be manipulated to facilitate the change of a community to a desired state. Studying the factors that affect keystone species and their influence on the environment can provide knowledge on the type of changes necessary for successful ecological restoration and how restoration efforts can be most effectively applied through natural ecosystem engineering.

Algae have frequently been shown to provide protection to epifauna against predation (Bueno & Leite, 2019; Bueno et al., 2020; Lanham et al., 2020; Ware et al., 2019), but in this system, we found no evidence of direct top‐down control of epifaunal numbers by predators. On the contrary, we did find evidence of both top‐down (grazing) and bottom‐up (nutrient supply) effects on macroalgae, which had indirect effects on epifauna by modifying their habitat.

We compared the strength of these direct and indirect effects by manipulating grazing and predation pressure at sites experiencing either frequent or infrequent upwelling events. We rejected our predictions that predation would have significant effects at all sites and that this effect would be particularly strong where algal growth is not promoted by upwelling. Similarly, we rejected our hypothesis that upwelling would have an indirect effect on epifauna. Lastly, we conclude that grazing has a significant indirect effect on the epifauna when epifaunal densities were normalized to algal cover. To carry out our experiment, we characterized sites a priori as strongly or weakly influenced by upwelling, using this as a proxy for the degree of nutrient supply. We then tested our initial characterization by estimating the intensity, duration, and intensity of upwelling at each site. Many studies have successfully used a rapid drop in water temperature as a measure of the frequency of coastal upwelling (e.g., while data on hourly wind speed and direction have been used to calculate an upwelling index by, allowing the estimation of not just the frequency of upwelling events, but also their duration and intensity. We used both approaches, using sea surface temperature (SST) to calculate the number of upwelling events and wind data (speed and direction) to confirm upwelling frequency and estimate the intensity and duration of events, and distinguishing upwelling events of short, medium, and long duration. The results from both data sets confirmed our a priori classification of shores by upwelling (i.e., Port Alfred and Brenton‐on‐Sea exhibited more upwelling events and upwelling days), though, unexpectedly, Brenton‐on‐Sea had markedly lower upwelling indices than the other sites.

### 4.1 Algal responses

We expected upwelling to affect the results of grazing by altering the balance between algal growth and removal. In this case, upwelling did indeed interact with grazing, but counterintuitively, effects of grazer exclusion were significant only at upwelling sites. Increased nutrient supply due to upwelling is usually associated with enhanced local primary production and algal standing stocks (Xavier et al., 2007), but this was not the case in the present experiment, with upwelling sites having roughly the same percentage algal cover as non‐upwelling sites at both the start and end of the experiment. By the end of the experiment, treatments that allowed access to grazers (i.e., Grazer+ and Control) had similar levels of algal cover at both upwelling and non‐upwelling sites and this may be attributed to the fact that there were roughly twice as many grazers at upwelling sites (AN unpub. data). This presumably results in greater grazing pressure and cancels out the positive effects of upwelling. In this case, upwelling did indeed interact with grazing, but counterintuitively, the effects of grazer exclusion were significant only at upwelling sites where higher numbers of grazers were more than compensated for (presumably) higher algal growth rates.

Algal cover in all treatments showed strong seasonality at all sites, declining through the winter months (June–August) and increasing through spring and summer. The seasonal pattern was, however, modified where grazers had access and treatments that allowed grazing showed much greater intersite variation during the course of the year. This modification within treatments that allowed access to grazers indicates the overriding of seasonal variation by top‐down forcing.

Strong effects of treatment on algal cover (Figure 4) indicated strong grazing effects, with plots that excluded grazers (Closed and Predator+) having significantly greater cover than plots allowing access to grazers (Grazer+ and Controls). The interaction between upwelling and treatment was significant, but this reflected a difference in the intensity of this pattern, not a difference in the pattern. At both types of sites, grazed plots had less algal cover, but the Predator+ vs Grazer+ and Closed vs Control comparisons were nonsignificant at one of the non‐upwelling sites and thus the pooled data, leading to an interaction.

Critically, grazing also affected the structural complexity of algae. Because of its complex ramified structure, the habitable volume of a tuft of macroalga is not equivalent to the three‐dimensional space in which it resides. Instead, it is defined by the complex spacing between thalli, and its fractal dimension, *D*, lying between *D* = 2 and *D* = 3, is a measure of the degree to which space is filled. The disproportionate increase in surface area with decreasing scales results in more usable space for the associated epifauna (Gee & Warwick, 1994a, 1994b; Gunnarsson, 1992; Lawton, 1986; Morse et al., 1985; Shorrocks et al., 1991).

Algae at the study sites are subject to grazing not only by benthic grazers but also by herbivorous fish (e.g., Götz et al., 2009; Heemstra & Heemstra, 2004) that would not have been excluded by our unroofed fences. Thus, Control plots will have suffered both benthic and pelagic grazing, leading to a decline in structural complexity and minimal values of D. Grazer+ plots were roofed, suffering only benthic grazing, while the Predator+ plots would have suffered only pelagic grazing, probably with some inhibition of fish behavior due to the fences. Consequently, there was an effective gradient in grazing intensity among our treatments that was reflected in the results of the fractal analysis.

### 4.2 Epifauna

The community structure of epifaunal communities, based on species abundances, showed no effects of either upwelling or treatment, but did show a significant effect of site, with all sites differing from one another. This appears to have been a geographic effect as sites that were closer together were more similar. Importantly, while total epifaunal abundance responded only to the effects of site, when the data were normalized for habitat availability there were significant treatment effects. These effects indicated a response of the epifauna to indirect effects on their habitat, but not to the direct effects of predation. The important result here was the contrast between treatment effects on epifaunal abundances when these were normalized to algal cover (Table 5), indicating that grazing has important indirect effects on epifaunal abundances through its influence on habitat availability.

**TABLE 5:** Tukey's HSD summary of results for the combined data normalized for algal cover

	Closed	Control	Grazer +	Predator +
Closed		**0.006**	**0.001**	0.999
Control			0.927	**0.004**
Grazer +				**0.001**
Predator +				

Note

Significant results (*p* < .05) in bold.

The direct advantages to epifauna of using algae as protection from predation and the importance of algal morphology are well known (e.g., Cacabelos et al., 2010), though in our case, predation did not influence the epifauna significantly, but indirect effects and how grazing can influence epifauna by altering habitat quality have not been explored. The shape and structure of macroalgae are important determinants of the abundance and size of associated epifauna with more structurally complex algae providing habitat to abundant and diverse epifauna (Reynolds et al., 2014; Williams et al., 2013). Invertebrate grazers relying on macroalgae as food have strong direct effects on algae, including altering not only their abundance but also their structure (Cook et al., 2011; Reynolds et al., 2014). In our experiments, grazing had a significant influence on the fractal dimensions of algae, indicating a change in structural complexity. Our results indicate that this in turn affects the epifauna by altering the architectural quality of their habitat.

Neither grazing nor predation significantly affected total epifaunal abundances, but a reduction in algal cover and complexity by grazers resulted in increased epifaunal densities in the presence of grazers. Thus, epifaunal densities were maintained not by the direct effects of predation or grazing, but by indirect effects of grazers on algal structure. This accords with frequent observation that epifaunal numbers are related not to algal species identity, but to their structural complexity (Gan et al., 2019; Lutz et al., 2019; Veiga et al., 2016). As a result, we conclude that epifauna respond to the indirect effects of top‐down regulation of their habitat, rather than to the bottom‐up effects of nutrient availability, or the direct top‐down effects of predation. Understanding the processes that determine the abundance, distribution, and persistence of ecosystem engineers and their effects on the environment is of paramount importance as it enables the management of diverse ecological communities. It is not only the effect of ecosystem engineers on their environment that is important but also the factors (in this case, nutrient availability and grazing) that drive their population dynamics as this can have cascading effects on the whole community. Our results show how factors that influence an ecological engineer (in this case, grazing and nutrient supply) can affect the quality of habitat that it offers, with powerful indirect consequences for dependent communities.

## CONFLICT OF INTEREST

The authors declare that they have no conflict of interest.

## AUTHOR CONTRIBUTIONS

**Aldwin Ndhlovu:** Data curation (equal); Formal analysis (equal); Investigation (equal); Methodology (equal); Validation (equal); Visualization (equal); Writing‐original draft (equal); Writing‐review & editing (equal). **Justin A. Lathlean:** Conceptualization (equal); Data curation (equal); Formal analysis (equal); Methodology (equal); Software (equal); Supervision (equal); Validation (equal); Visualization (equal); Writing‐review & editing (equal). **Christopher D. McQuaid:** Conceptualization (equal); Data curation (equal); Formal analysis (equal); Funding acquisition (equal); Methodology (equal); Supervision (equal); Validation (equal); Visualization (equal); Writing‐original draft (equal); Writing‐review & editing (equal). **Laurent Seuront:** Formal analysis (equal); Investigation (equal); Methodology (equal); Software (equal); Validation (equal); Visualization (equal); Writing‐review & editing (equal).

## Data Availability

The data sets generated and/or analyzed during the current study are available in the Dryad repository: https://doi.org/10.5061/dryad.931zcrjkz.

## 1

**TABLE A1:** Species identified in this study

Amphipods	Isopods	Gastropods	Polychaetes
*Afrochiltonia capensis*	*Cymodecella sublevis*	*Burnupena lagenaria*	*Boccardia polybranchia*
*Amaryllis macrophthalma*	*Dynamenella australis*	*Burnupena pubescens*	*Eunice aphroditois*
*Ampelisca palmata*	*Dynamenella huttoni*	*Helcion dunkeri*	*Lepidonotus semitectus clava*
*Atylus swammerdamei*	*Exosphaeroma pallidum*	*Gibbula multicolor*	*Lumbrineris tetrauna*
*Hyale grandicornis*	*Tylos capensis*	*Oxystele variegata*	*Scololepis squamata*
*Leucothoe spinicarpa*	*Jaeropsis paulensis*	*Siphonaria concinna*	*Naineris laevigata*
*Lysianassa ceratina*	*Synidotea variegata*	*Tricolia capensis*	*Notomastus latericeus*
*Metaleptamphopus membrisetata*	*Exosphaeroma porrectum*	*Tricolia neritina*	*Pseudonereis variegata*
*Paramoera capensis*	*Isopoda species A*	*Gastropoda species A*	*Lysidice natalensis*
*Parandania boecki*	*Isopoda species B*		*Arabella iricolor*
*Phistica marina*		**Bivalves**	*Polychaeta species A*
*Nicippe tumida*	**Chitons**	*Mytilus galloprovincialis*	*Polychaeta species B*
*Amphipoda species A*	*Onithochiton literatus*	*Perna perna*	
	*Acanthochiton garnoti*	*Choromytilus meridionalis*	
	*Chitonidae species A*		
